# Romanticism, Mycobacterium, and the Myth of the Muse

**DOI:** 10.3201/eid2503.AC2503

**Published:** 2019-03

**Authors:** Dennis Mahoney, Terence Chorba

**Affiliations:** University of Vermont, Burlington, Vermont, USA (D. Mahoney);; Centers for Disease Control and Prevention, Atlanta, Georgia, USA (T. Chorba)

**Keywords:** art science connection, emerging infectious diseases, art and medicine, about the cover, Mycobacterium, tuberculosis, public health, bacteria, romanticism, Mycobacterium, and the myth of the muse, tuberculosis and other mycobacteria, Georg Philipp Friedrich von Hardenberg (Novalis)

**Figure:**
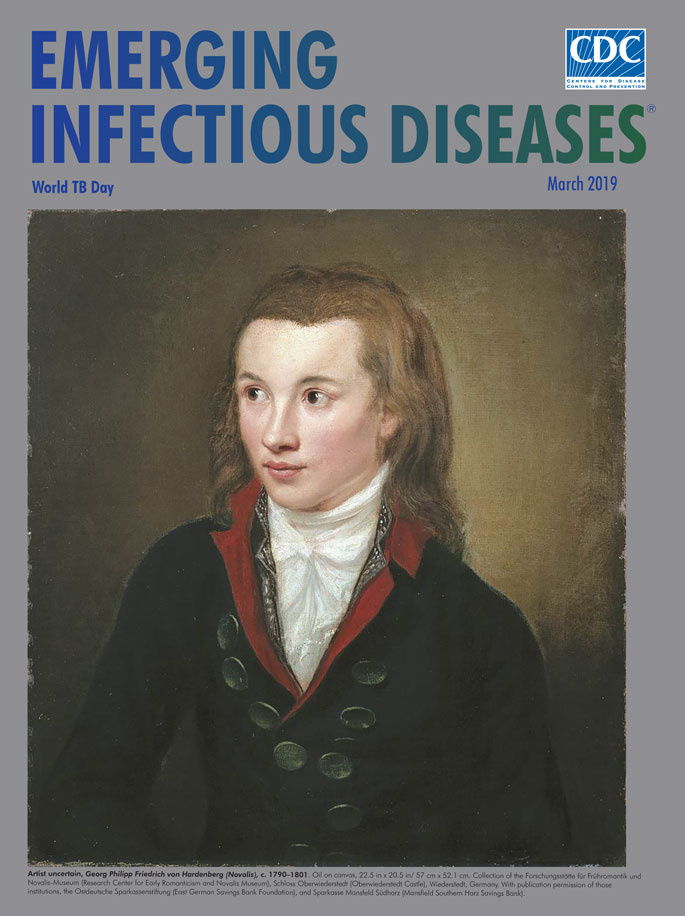
**Artist uncertain, Georg Philipp Friedrich von Hardenberg (Novalis), c. 1772–1801.** Oil on canvas, 22.5 in x 20.5 in/57 cm x 52.1 cm. Collection of the *Forschungsstätte für Frühromantik und Novalis-Museum* (Research Center for Early Romanticism and Novalis Museum), *Schloss Oberwiederstedt* (Oberwiederstedt Castle), Wiederstedt, Germany. With publication permission of those institutions, the *Ostdeutsche Sparkassenstiftung* (East German Savings Bank Foundation), and *Sparkasse Mansfeld Südharz* (Mansfield Southern Harz Savings Bank).

At the transition of the 18th into the 19th century, large numbers of deaths in Europe, especially those in urban areas, were associated with tuberculosis. During those two centuries, many celebrated artists, musicians, and literary giants were lost to the disease. Romanticism*—*Europe’s dominant artistic, musical, and intellectual movement that began in the late 18th century and waned after 1850*—*emphasized individualism and emotion. Characteristic themes included the goodness of people, from which urban life detracted, and the simplicities of childhood and all things natural. A popular myth arose that this movement was favored by tuberculosis, which putatively augmented one’s creative faculties. Classicists viewed this belief as consistent with what ancient Greek physicians had called the *spes phthisica—*an earnest hope of recovery from tuberculosis that drove heightened sensitivity and great creativity despite overwhelming illness. Portrayals of this view appear in Alexander Dumas's *La Dame aux Camélias*, Victor Hugo's *Les Misérables*, Giuseppe Verdi's *La traviata*, and Giacomo Puccini's *La bohème.*

Among German writers of the Romantic era who had tuberculosis were Johann Wolfgang von Goethe (1749–1832; best known to English speakers for his poetic drama *Faust*), Friedrich Schiller (1759–1805; trained as a physician and author of “An die Freude”—the Ode to Joy in the final movement of Beethoven’s Ninth Symphony), and Georg Philipp Friedrich von Hardenberg (1772–1801; principal poet-theoretician of Early German Romanticism). Goethe received his tuberculosis diagnosis when in his early 20s and recovered fully after several years of convalescence. In contrast, Schiller died of pulmonary tuberculosis at age 46 after a period of increasing lethargy.

The undated and unsigned portrait on this month’s cover is that of Hardenberg, better known under his pen name Novalis (i.e., “the clearer of new ground,” a poetic goal derived from the surname of his medieval ancestors). Beginning in 1790, he studied at the University of Jena under Schiller and later completed his doctorate in law at the University of Wittenberg, both in what is modern-day Germany. After losing his first fiancée, Sophie von Kühn, to tuberculosis in 1797, he studied mining in Freiberg and fell in love with Julie von Charpentier, daughter of one of his professors. In August 1800, they intended to marry, but he began coughing up blood. His worsening health led him to return to Jena for a consultation with Johann Christian Stark, the physician who had provided treatment for Schiller and Sophie von Kühn. After unsuccessful treatment in Dresden, in January 1801 Hardenberg returned to the family residence of Weissenfels in Saxony (Figure), where he died on March 25. At his deathbed was Friedrich Schlegel (1772–1829), who had already published *Hymnen an die Nacht* (Hymns to the Night), a unique combination of rhythmic prose and strophic verse that helped establish Hardenberg's reputation as a poet. Schlegel also published a collection of Hardenberg’s poetic writings after Hardenberg died, co-edited by Ludwig Tieck (1773–1853), another leading figure in German Romanticism. This collection included *Heinrich von Ofterdingen,* an eponymous piece of historical fiction, one of the best-known and most uncompromising depictions of the transformative power of art and the metaphysical quest for the unattainable.

**Figure:**
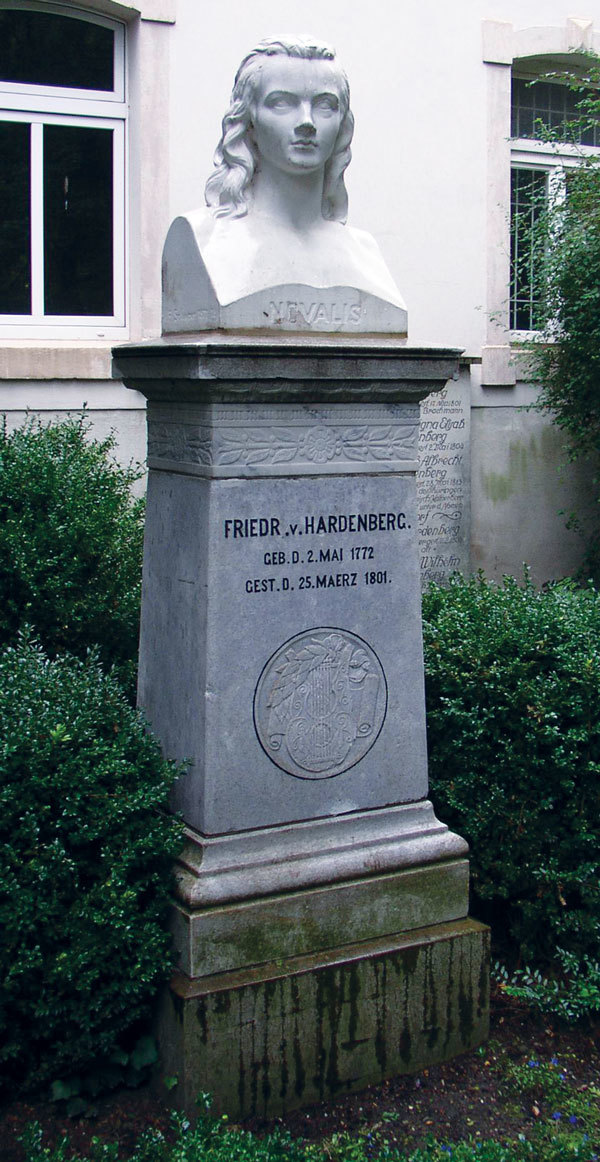
Memorial at the grave of Novalis in Weissenfels with a bust of the poet created by Fritz Schaper, a renowned sculptor and member of the Prussian Academy of the Arts, in 1901. Photograph by Doris Antony, Berlin, Germany.

Until recently, this cover portrait was widely attributed to Franz Gareis (1775–1803). Gareis was a student of the noted portrait painter Anton Graff, who had painted a portrait of Schlegel in 1798. Later, Schlegel asked Hardenberg to be the subject of a Gareis portrait, but there is no indication that Gareis and Hardenberg ever met. A more likely creator of this portrait was Tieck’s sister-in-law, Maria Agatha Alberti (1767–1810), a private student of Graff and Gareis in Dresden at a time when women were not admitted for study at the Dresden Art Academy. Alberti was also part of the circle of friends who ministered to Hardenberg in his final winter. In a letter in June 1801, Karl von Hardenberg, the poet’s brother, asked of Tieck that Alberti paint the poet posthumously. Recently published letters show that in 1805, Alberti was in Weissenfels, where she made portraits of Karl, his mother, and his nephew Erasmus. If the Novalis portrait is by Alberti, it is a retrospective depiction, perhaps based on an earlier sketch of the poet, conveying both his frailty and immortal spirit. Tieck attested to the authenticity of this portrait and asked the Hardenberg family whether he might keep it in his possession until his death in 1853.

Several years later, Jean-Antoine Villemin demonstrated the transmissibility of tuberculosis by infecting laboratory rats with material extracted from human cadavers. In 1882, Robert Koch identified *Mycobacterium tuberculosis* by staining the sputum of patients with pulmonary tuberculosis with alkaline methylene blue; on March 24 of that same year, a generation after Romanticism began to wane, he demonstrated conclusively that *M. tuberculosis* was the causative organism of the disease. A societal change of view then followed; the Romanticist position that consumption was a tragic gift inspiring creativity diminished. By the end of the 19th century, the dominant view was that environmental controls and social distancing benefited the community by providing protection from this contagious illness. The result was dramatically decreased numbers of cases of illness and death from tuberculosis, several decades before effective antimicrobials became available.
